# Identification of Clinically Relevant *Streptococcus* and *Enterococcus* Species Based on Biochemical Methods and 16S rRNA, *sodA*, *tuf*, *rpoB*, and *recA* Gene Sequencing

**DOI:** 10.3390/pathogens9110939

**Published:** 2020-11-11

**Authors:** Maja Kosecka-Strojek, Mariola Wolska, Dorota Żabicka, Ewa Sadowy, Jacek Międzobrodzki

**Affiliations:** 1 Department of Microbiology, Faculty of Biochemistry, Biophysics and Biotechnology, Jagiellonian University, 30-387 Krakow, Poland; mariola.wolska@doctoral.uj.edu.pl (M.W.); jacek.miedzobrodzki@uj.edu.pl (J.M.); 2 Department of Molecular Microbiology, National Medicines Institute, 00-725 Warsaw, Poland; d.zabicka@nil.gov.pl; 3 Department of Epidemiology and Clinical Microbiology, National Medicines Institute, 00-725 Warsaw, Poland; e.sadowy@nil.gov.pl

**Keywords:** streptococci, enterococci, molecular diagnostics, genetic methods, Sanger sequencing

## Abstract

Streptococci and enterococci are significant opportunistic pathogens in epidemiology and infectious medicine. High genetic and taxonomic similarities and several reclassifications within genera are the most challenging in species identification. The aim of this study was to identify *Streptococcus* and *Enterococcus* species using genetic and phenotypic methods and to determine the most discriminatory identification method. Thirty strains recovered from clinical samples representing 15 streptococcal species, five enterococcal species, and four nonstreptococcal species were subjected to bacterial identification by the Vitek^®^ 2 system and Sanger-based sequencing methods targeting the 16S rRNA, *sodA*, *tuf*, *rpoB*, and *recA* genes. Phenotypic methods allowed the identification of 10 streptococcal strains, five enterococcal strains, and four nonstreptococcal strains (*Leuconostoc*, *Granulicatella*, and *Globicatella* genera). The combination of sequencing methods allowed the identification of 21 streptococcal strains, five enterococcal strains, and four nonstreptococcal strains. The 16S rRNA and *rpoB* genes had the highest identification potential. Only a combination of several molecular methods was sufficient for unambiguous confirmation of species identity. This study will be useful for comparison of several identification methods, both those used as a first choice in routine microbiology and those used for final confirmation.

## 1. Introduction

Gram-positive bacteria of the *Streptococcus* and *Enterococcus* genera are of great clinical and epidemiological importance, and most species are components of the natural human microbiota [1]. The genus *Streptococcus* includes a large number (at least 135) [2,3] [https://www.bacterio.net/genus/streptococcus] of species that colonize human and animal mucous membranes. Species such as *Streptococcus pyogenes*, *Streptococcus pneumoniae*, and *Streptococcus agalactiae* are highly virulent and cause infections and diseases such as scarlet and rheumatic fevers, pneumonia or neonatal sepsis [4,5,6]. Streptococci are classified based on colony morphology, hemolysis type, and serological specificity. The serological specificity is based on antigenic differences in cell wall carbohydrates, in cell wall pilus-associated proteins, and in the polysaccharide capsule in group B streptococci [7]. The classification and nomenclature of streptococci are based on group antigens (Lancefield serotyping system) as follows: group A *Streptococcus* (GAS); group B *Streptococcus* (GBS); group C *Streptococcus*; group G *Streptococcus*; the viridans group, with the subgroups anginosus, mitis, mutans, and salivarius; and the bovis group [8,9,10].

The members of the genus now known as *Enterococcus* were formerly considered to be group D *Streptococcus* until 1984 [11]. Isolates from the *Enterococcus* genus are commensals of the gastrointestinal tracts of humans and animals and include 64 species [12,13] [https://bacterio.net/genus/enterococcus]. All *Enterococcus* species are classified into the antigen D group by the Lancefield system [11] and exhibit gamma-hemolysis on blood agar, although some strains are alpha-hemolytic or beta-hemolytic [14,15]. *Enterococcus faecalis* and *Enterococcus faecium* can cause a variety of infections, including endocarditis and urinary tract infections [16,17].

The addition of new species, changing taxonomy and modification of the systematic names of streptococci and enterococci, poses a challenge to proper identification of species. Therefore, precise identification of these species is laborious. Clinical laboratories use phenotypic biochemical methods such as Vitek^®^ 2 (bioMérieux, La Balme Les Grottes, France) and BD Phoenix (BD Diagnostic Systems, Sparks, MD, USA), commercial rapid test kits such as API^®^ Strep (bioMérieux, La Balme Les Grottes, France) and matrix-assisted laser desorption ionization–time-of-flight mass spectrometry (MALDI-TOF MS). In routine diagnostics, especially the Vitek^®^ 2 system is used. This system is based on kinetic analysis detecting metabolic changes and by additional continuous monitoring of reactions, provides much faster species identifications [18]. Nevertheless, the technique so far has failed at differentiating between mitis, bovis groups, and other closely relative species [19,20]. On the other hand, commercially available MALDI-TOF MS systems provide accurate identification of many clinically relevant streptococcal species. However, MALDI-TOF spectra databases are limited to only some species, and further improvements of *Streptococcus* and *Enterococcus* spectra databases seem necessary. The phenotypic trait variability within strains and species using this method compared to methods based on genetic discrimination causes limited differentiation capacity; consequently, more than 50% of these bacteria are incorrectly identified [21,22].

The development of molecular biological techniques has made it possible to rapidly and reliably diagnose infections caused by bacteria of the *Streptococcus* and *Enterococcus* genera. Genetic methods are based on PCR or sequencing, and identification is based on selected molecular target amplification, sequencing, and comparison to a reference sequence deposited in a nucleotide database [13]. 16S rRNA gene sequencing has proven to be one of the most powerful tools for the classification of microorganisms, including streptococci and enterococci [1,23]. However, due to low specificity, the correct identification of bacterial species should not be based on the nucleotide sequence of a single gene. For unambiguous species confirmation, it is necessary to use additional molecular markers. For the identification of *Streptococcus* and *Enterococcus* isolates, several gene targets, such as genes encoding manganese-dependent superoxide dismutase (*sodA*) [24], the elongation factor Tu (*tuf*) [25], and beta-subunit of RNA polymerase (*rpoB*) [26], have been used. Furthermore, for species included in the mitis (currently includes about 20 different species [27,28]) and bovis (*Streptococcus bovis*, *Streptococcus equinus*, *Streptococcus gallolyticus*, *Streptococcus lutetiensis*, *Streptococcus alactolyticus* [29]) groups, which are closely related, other conserved molecular targets, such as the subunit of the bacterial recombinase (*recA*) gene, may be used [30,31].

The aim of this study was to identify clinically relevant *Streptococcus* and *Enterococcus* species using genetic and phenotypic methods and to determine the most discriminatory identification method. In our study, the Vitek^®^ 2 system and Sanger sequencing of five genes, namely, the 16S rRNA, *sodA*, *tuf*, *rpoB*, and *recA* genes, were used.

## 2. Results

### 2.1. Serotyping and Identification of Gram-Positive Cocci with the Vitek^®^ 2 System and MALDI-TOF MS

After recovering the isolates from clinical samples, the hospital laboratories identified all of the isolates at the genus level. All isolates were identified as *Streptococcus* and *Enterococcus* with routine diagnostic methods. Afterwards, serotyping and identification at the species level were performed in our laboratory. The Lancefield serotype groups were assigned: 57% streptococci, 60% enterococci, and 50% other nonstreptococci. No visible agglutination of latex or autoagglutination with more than one reagent with antibody particles was interpreted as ambiguous. Briefly, in the streptococcal serotype identification performed with the Pastorex™ Strep Test Kit (Bio-Rad, Hercules, CA, USA), a positive reaction is indicated by red clumps on a green background, visible to the naked eye. Agglutination intensity and time of appearance depend upon the strain tested. Only marked, rapid agglutination with only one of the six latex suspensions convincingly establishes the group of the strain tested. A negative reaction is indicated by a homogenous brown suspension, without clumps, after one minute of agitation. A reaction is un-interpretable if small clumps appear on a brown background, or if agglutination appears with more than one latex reagent in the kit [32].

The Vitek^®^ 2 system allowed for identification of 10 of the 21 *Streptococcus* strains, all five *Enterococcus* strains, and three nonstreptococcal strains (*Globicatella sanguinis*, *Leuconostoc lactis*, and *Leuconostoc citreum*) (Table 1). The Vitek^®^ 2 procedure and serotyping were performed for all isolates, and MALDI-TOF MS was performed for ambiguous and untypable isolates. Most streptococci and enterococci species were identified at excellent (67% *Streptococcus*; 40% *Enterococcus*) and very good (14% *Streptococcus*; 60% *Enterococcus*) discrimination levels. For 29% of the streptococcal strains (PL427, S63, 1816/15, 1226/14, PL431 1374/11), the Vitek^®^ 2 system did not allow identification at the species level, and only the indistinguishable *S. mitis* or *S. oralis* group was assigned. The strains 6922/09 and 1860/08 were assigned as *Streptococcus anginosus/Streptococcus gordonii* and *Streptococcus agalactiae/Streptococcus dysgalactiae*, respectively. The PL434 strain was identified as *Kocuria rosea*, and p41 was not identified at all.

For isolates which were not identified as *Streptococcus* or *Enterococcus* by the Vitek^®^ 2 system (PL434, 1113/11, 3696/08, p41, and 1375/11), MALDI-TOF MS was used. All isolates were identified with a high degree of confidence (≥2.00). For the strains 1113/11, 3696/08, and 1375/11, MALDI-TOF MS showed the same identification results as the Vitek^®^ 2 system. In the case of PL434 (identified as *Kocuria rosea* by Vitek^®^ 2), MALDI-TOF MS identified this isolate as *Granulicatella adiacens*, and p41 was identified as *S. pneumoniae* (Table 1).

### 2.2. Sanger Sequencing of the 16S rRNA Gene

Sanger sequencing of the 16S rRNA gene allowed identification of 19 *Streptococcus* strains (90% of all streptococcal strains), four *Enterococcus* strains (80% of all enterococcal strains), and three of the four nonstreptococcal strains (*G. adiacens*, *G. sanguinis*, *L. citreum*). Identification of the following pairs of enterococcal, streptococcal, and nonstreptococcal species was impossible because the 16S rRNA gene sequences were identical or almost identical (≥99.8% identity): *Enterococcus raffinosus/Enterococcus gilvus; Streptococcus australis/Streptococcus sanguinis; S. pneumoniae/S. mitis; L. lactis/Leuconostoc garlicum* (Table 2).

### 2.3. Sanger Sequencing of the sodA Gene

Sanger sequencing of the *sodA* gene allowed identification of 12 *Streptococcus* strains (57% of all streptococcal strains), four *Enterococcus* strains (80% of all enterococcal strains), and two of the four nonstreptococcal strains (*G. adiacens*, *G. sanguinis*). Identification of the following pairs of enterococcal, streptococcal, and nonstreptococcal species was impossible because the *sodA* gene sequences were identical or almost identical (≥99.8% identity): *E. faecalis/E. faecium; S. anginosus/Streptococcus milleri; Streptococcus lutetiensis/Streptococcus infantarius; Streptococcus parasanguinis/S. oralis; S. mitis/Streptococcus cristatus; S. pyogenes/S. dysgalactiae; L. citreum/S. parasanguinis.* For strain 1113/11 (*L. lactis*), there was no *sodA* gene reference sequence in any database (Table 2).

### 2.4. Sanger Sequencing of the tuf Gene

Sanger sequencing of the *tuf* gene allowed identification of 13 *Streptococcus* strains (62% of all streptococcal strains), five *Enterococcus* strains (100% of all enterococcal strains), and three of the four nonstreptococcal strains (*G. adiacens*, *G. sanguinis*, *L. citreum*). Identification of the following pairs of enterococcal, streptococcal, and nonstreptococcal species was impossible because the *tuf* gene sequences were identical or almost identical (≥99.8% identity): *S. anginosus/S. milleri; S. infantis/S. oralis; S. lutetiensis/S. infantarius; S. oralis/S. infantarius; S. pneumoniae/S. mitis; L. lactis/L. garlicum* (Table 2).

### 2.5. Sanger Sequencing of the rpoB Gene

Sanger sequencing of the *rpoB* gene allowed identification of 18 *Streptococcus* strains (86% of all streptococcal strains), five *Enterococcus* strains (100% of all enterococcal strains), and all four nonstreptococcal strains (*G. adiacens*, *G. sanguinis*, *L. lactis*, *L. citreum*). Identification of the following pairs of enterococcal, streptococcal, and nonstreptococcal species was impossible because the *rpoB* gene sequences were identical or almost identical (≥99.8% identity): *S. anginosus/Streptococcus intermedius; S australis/S. infantis; S. pseudopneumoniae/S. mitis* (Table 2).

### 2.6. Analysis of the recA Gene for the Streptococcal mitis Group

The streptococcal species that belong to the mitis group (*S. pneumoniae*, *S. pseudopneumoniae*, *S. mitis*, *S. oralis*, *S. gordonii*, *S. sanguinis*, and *S. parasanguinis*) are closely related phylogenetically.

**Figure 1:**
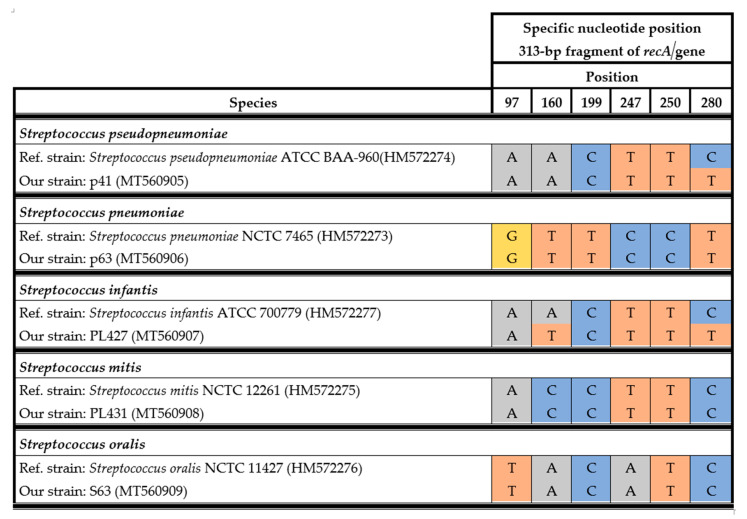
Alignment of specific nucleotides for the streptococcal mitis complex observed in the 313-bp *recA* fragment.

For precise differentiation of species within this group, sequencing of the *recA* gene was used. The specific nucleotide signatures of the 313-bp fragment of the *recA* gene sequence were compared to reference sequences in GenBank (HM572273–HM572277). Sanger sequencing of the *recA* gene allowed precise identification of strains from the mitis group, namely, *S. pneumoniae*, *S. pseudopneumoniae*, *S. mitis*, *S. oralis*, and *S. infantis*. The alignment showed six specific nucleotides at positions 97, 160, 199, 247, 250, and 280 (Figure 1). The nucleotide signature is based on homology analyses of *recA* gene sequences from reference strains of the aforementioned species and our strains. The *recA* gene sequence of the p41 strain was almost identical to the reference sequence (*S. pseudopneumoniae*), with a one-nucleotide difference at position 280. For PL427, differences at two nucleotide positions were observed in comparison to *S. infantis.* The only method that allowed unambiguous identification of *S. pseudopneumoniae* was Sanger sequencing of the *recA* gene.

### 2.7. Comparison of the Sequencing Methods

The combination of sequencing methods based on the 16S rRNA, *sodA*, *tuf*, *rpoB*, and *recA* genes allowed the identification of 21 streptococcal strains, five enterococcal strains, two *Leuconostoc* strains, one *Globicatella sanguinis* strain, and one *Granulicatella adiacens* strain. Due to high (or identical) similarity or a lack of similarity with the reference sequences in GenBank and leBIBI^QBPP^, it was not possible to identify all the strains at the species level by using the targets separately (Table 2).

For *Streptococcus*, Sanger sequencing of the 16S rRNA gene had the highest identification potential, allowing the identification of 19 (90%) strains. Additionally, *rpoB* gene sequencing had high discriminative potential, allowing the identification of 18 (86%) *Streptococcus* strains. Sanger sequencing of the *tuf* gene had moderate identification potential and identified 13 (62%) streptococcal strains. Sanger sequencing of the *sodA* gene had the lowest discriminatory potential, allowing the identification of 12 (57%) streptococcal strains.

Sanger sequencing of *rpoB* and *tuf* allowed the identification of five (100%) analyzed enterococcal strains. Sequencing of the 16S rRNA and *sodA* genes had moderate identification potential and allowed the identification of four (80%) enterococcal strains (Table 3).

### 2.8. Phylogenetic Analysis of Streptococcus and Enterococcus

**Figure 2:**
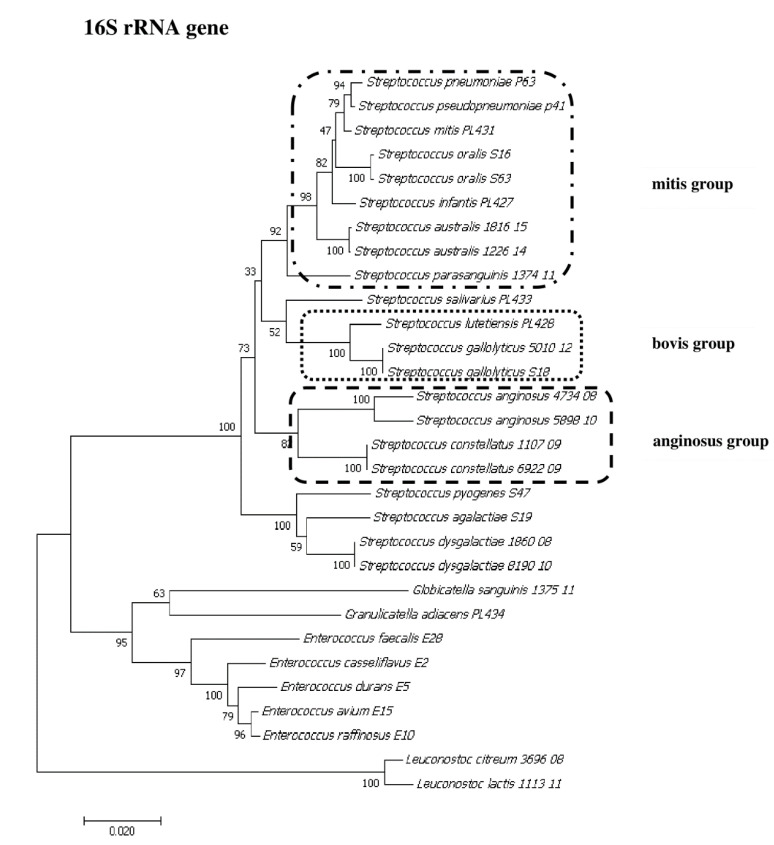
The phylogenetic tree of streptococcal and enterococcal species evolutionary relationships based on the 16S rRNA gene. The phylogenetic trees were constructed using the neighbor-joining method. The percentage of replicate trees in which the associated taxa clustered together in the bootstrap test (1000 replicates) is shown next to the branches. The tree is drawn to scale, with branch lengths in the same units as those of the evolutionary distances used to infer the phylogenetic tree. The evolutionary distances were computed using the Jukes–Cantor method and are in the units of the number of base substitutions per site. The strains which are placed in boxes have grouped together in all methods used. The length of the compared sequences was 1296 bp.

**Figure 3:**
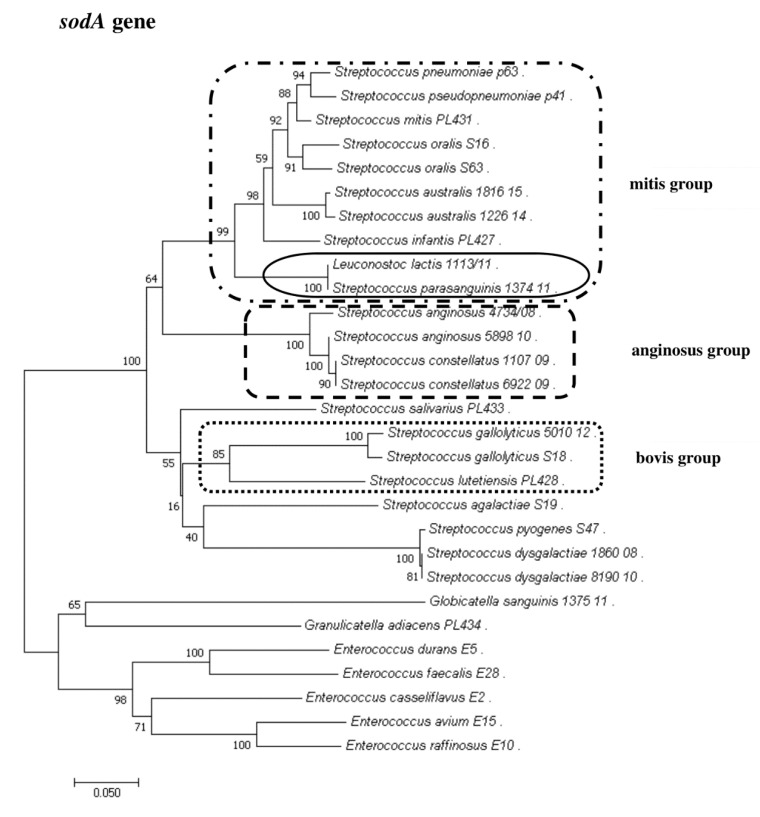
The phylogenetic tree of streptococcal and enterococcal species evolutionary relationships based on the *sodA* gene. The phylogenetic trees were constructed using the neighbor-joining method. The percentage of replicate trees in which the associated taxa clustered together in the bootstrap test (1000 replicates) is shown next to the branches. The tree is drawn to scale, with branch lengths in the same units as those of the evolutionary distances used to infer the phylogenetic tree. The evolutionary distances were computed using the Jukes–Cantor method and are in the units of the number of base substitutions per site. The strains which are placed in boxes have grouped together in all methods used. The length of the compared sequences was 418 bp.

**Figure 4:**
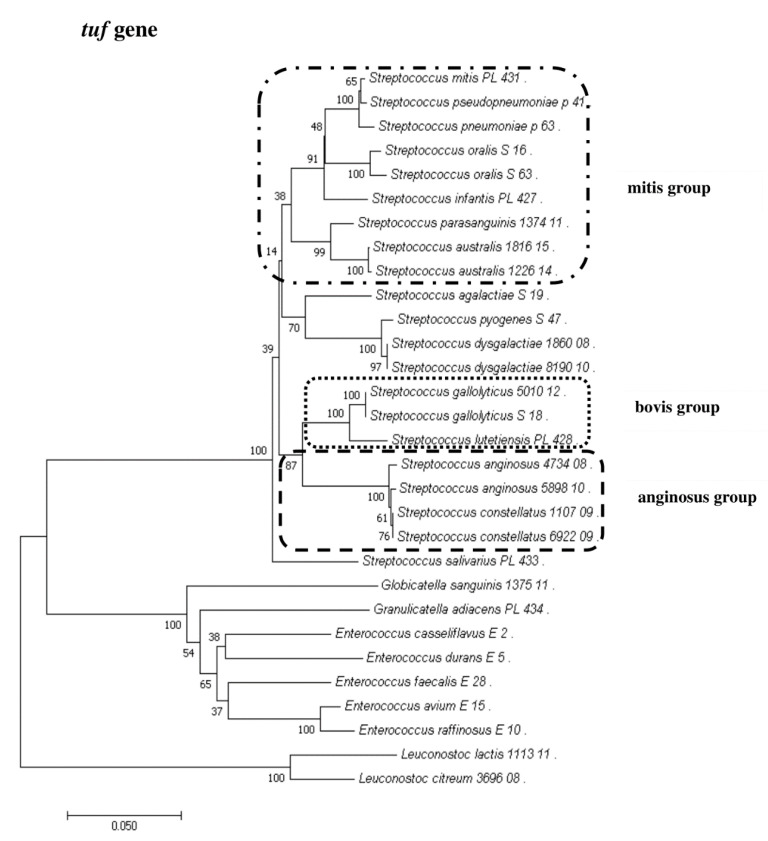
The phylogenetic tree of streptococcal and enterococcal species evolutionary relationships based on the *tuf* gene. The phylogenetic trees were constructed using the neighbor-joining method. The percentage of replicate trees in which the associated taxa clustered together in the bootstrap test (1000 replicates) is shown next to the branches. The tree is drawn to scale, with branch lengths in the same units as those of the evolutionary distances used to infer the phylogenetic tree. The evolutionary distances were computed using the Jukes–Cantor method and are in the units of the number of base substitutions per site. The strains which are placed in boxes have grouped together in all methods used. The length of the compared sequences was 770 bp.

**Figure 5:**
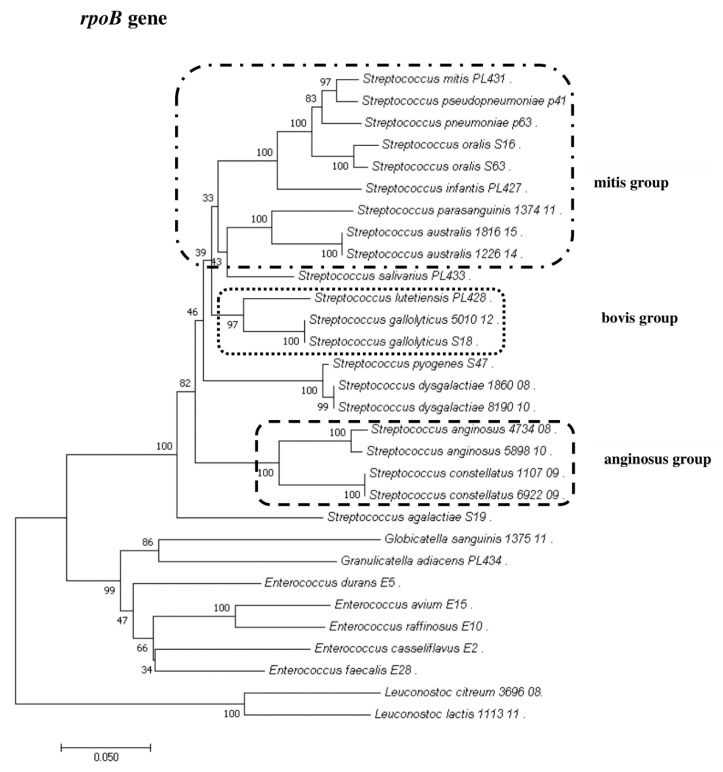
The phylogenetic tree of streptococcal and enterococcal species evolutionary relationships based on the *rpoB* gene. The phylogenetic trees were constructed using the neighbor-joining method. The percentage of replicate trees in which the associated taxa clustered together in the bootstrap test (1000 replicates) is shown next to the branches. The tree is drawn to scale, with branch lengths in the same units as those of the evolutionary distances used to infer the phylogenetic tree. The evolutionary distances were computed using the Jukes–Cantor method and are in the units of the number of base substitutions per site. The strains which are placed in boxes have grouped together in all methods used. The length of the compared sequences was 666 bp.

To show the relationships among the species, phylogenetic trees were constructed. The evolutionary distances were computed using the Jukes–Cantor method and are shown in units of the number of base substitutions per site. The computed overall means for the 16S rRNA, *rpoB*, *soda*, and *tuf* genes were 0.098, 0.225, 0.348, and 0.176, respectively. In the phylogenetic tree constructed for the *tuf* gene, the *Leuconostoc* species sequences are shorter because sequences of the same length as those of other species could not be obtained. Both streptococci and enterococci are grouped into separate clusters. Moreover, the *Streptococcus* strains are divided into mitis, bovis, and anginosus complexes. Sequencing of the 16S rRNA, *rpoB*, and *tuf* genes showed that *L. lactis*, *L. citreum*, *G. sanguinis*, and *G. adiacens* were distantly related to the other species (Figure 2, Figure 3, Figure 4 and Figure 5).

## 3. Discussion

Because of the variability of strains and challenging taxonomic changes of *Streptococcus* and *Enterococcus* species, it is necessary to use a reliable identification method to better understand the pathogenic potential of various streptococcal and enterococcal species. The currently used phenotypic identification methods based on morphological and biochemical characteristics appear to be unreliable and are characterized by low discriminatory potential [33,34,35].

In this study, we applied biochemical methods and genetic sequencing-based methods to identify clinically relevant *Streptococcus* and *Enterococcus* species. We showed that the Vitek^®^ 2 system and MALDI-TOF MS did not correctly identify particular closely related species, such as *S. mitis*, *S. oralis*, and other species of the mitis group. Overall, the phenotypic methods allowed the identification of 48% of streptococcal and 100% of enterococcal strains. These data are consistent with previous data in the literature [19,36,37,38,39]. Therefore, applying genetic methods in standard microbiological diagnostics can lead to unambiguous confirmation at the species level. Genotypic methods utilizing Sanger sequencing of targeted genes were shown to be useful for both *Streptococcus* and *Enterococcus* identification [13,25]. 16S rRNA is mostly used to identify unknown organisms because of the availability of universal primers [23,40]. However, most reports show that the discriminatory power of 16S rRNA gene sequencing is very low for closely related streptococcal and enterococcal species [13,41,42]. Analysis based on only one gene target is not recommended because duplication, gene transfer, and gene loss can affect the reliability of the results [43,44].

In this study, we used a combination of four gene targets (16S rRNA, *sodA*, *tuf*, *rpoB*) to unambiguously confirm the identity at the species level for 21 streptococci and five enterococcal strains. None of the individual sequencing-based methods allowed the identification of all species. In our study, Sanger sequencing of the 16S rRNA gene had the highest discriminatory power, allowing unambiguous identification of 19 (90%) of the analyzed streptococcal strains, but the *rpoB* gene had almost identical identification potential, allowing the identification of 18 (86%) *Streptococcus* strains. For *Enterococcus* strains, Sanger sequencing of the *tuf* and *rpoB* genes allowed the identification of five (100%) strains. The 16S rRNA and *sodA* genes did not allow identification of all *Enterococcus* strains, but in our study, this group was very small (only five strains).

Over the years, the taxonomy of bacteria has changed, and streptococcal groups, i.e., mitis and bovis, have undergone several reclassifications. Moreover, incorrect systematic names of bacteria have been deposited in publicly available databases [45]. In our study, several problematic situations occurred. First, *Streptococcus tigurinus* was classified as *S. oralis* subsp. *tigurinus*, but in 2012, this species was separated into two different species. Finally, in 2016, it was again proposed that this species be classified as *S. oralis* subsp. *tigurinus* [27,46]. Our sequence was aligned to the sequence of *S. oralis*, but the next closest species was *S. tigurinus*. Incorrect taxonomic annotations of DNA sequences are often present in databases [45]. A similar situation was found for *S. lutetiensis* (PL428 strain), which was described as *S. infantarius* subsp. *coli* based on the *sodA* and *tuf* genes. In 2005, the International Committee on Systematics of Prokaryotes (Status of strains that contravene Rules 27 (3) and 30 of the International Code of Nomenclature of Bacteria, Opinion 81) accepted *S. lutetiensis* as the correct systematic name [47], but in databases, double taxonomic annotation for one organism can be found.

The *Enterococcus* strain E28 (*E. faecalis*) *sodA* gene sequence matched *E. faecium* (412/412 nucleotide identity). In our study, such a situation did not occur for other gene targets, yet it has been reported in the literature [48,49]. On the other hand, for strain E10 *(E. raffinosus*), the 16S rRNA gene sequence matched two enterococcal species, namely, *E. gilvus* (1289/1290 nucleotide identity) and *E. raffinosus* (1289/1291 nucleotide identity).

For *Streptococcus*, there were also some problematic cases in the anginosus group (also known as the *S. milleri* group). Strain 5898/10 was identified as *S. anginosus* by 16S rRNA gene sequencing, but other molecular methods showed ambiguous identification among the *S. anginosus-S. milleri-S. intermedius* species. Such a situation was observed by others [50,51]. A similar problem was observed in the identification of the 1107/08 and 6922/09 strains. Only 16S rRNA and *rpoB* allowed *Streptococcus constellatus* identification, while for the *sodA* and *tuf* genes, our strain sequences shared high nucleotide similarities with both the *S. anginosus* and *S. milleri* sequences. The *Streptococcus milleri* group proved to be challenging to identify in previous studies [51,52].

Both phenotypic and genetic methods correctly identified the nonstreptococcal species as *Globicatella sanguinis*, *Granulicatella adiacens*, *Leuconostoc citreum*, and *Leuconostoc lactis*. *Globicatella sanguinis* was initially described as *Streptococcus uberis* and *Aerococcus viridans* due to similar phenotypic properties. The advanced methods allowed the distinguishing and classification of *G. sanguinis* into a new species [9,53,54,55]. In our study, this species was identified by all four gene targets (16S rRNA, *sodA*, *rpoB*, and *tuf*).

*Granulicatella adiacens* was first described as *Streptococcus adjacens* and then as belonging to the *Abiotrophia* genus due to distant relations with streptococci. Collins and Lawson proposed a new genus, *Granulicatella*, due to significant differences [56,57]. In our study, strain PL434 was identified as *G. adiacens* by all sequencing methods.

The *Leuconostoc* genus is often identified as *Streptococcus* spp. Because similar biochemical properties and serotypes of the D group are observed, *Leuconostoc* species are difficult to detect with routine diagnostic methods [9]. It has been suggested that *Leuconostoc* is a pathogen that colonizes the gastrointestinal tract and is present in neutropenic patients [58,59]. For the *Leuconostoc* genus, strain 1113/11 was correctly identified by the Vitek^®^ 2 system and based on the *rpoB* gene, but the 16S rRNA and *tuf* genes were ambiguous between *L. lactis* and *L. garlicum*. For the *sodA* gene, there was no *L. lactis* reference sequence available in databases, but the sequence was identical to *S. parasanguinis*. Such results were not observed by other research groups, but our study showed that in some cases the distinction between two bacterial genera is not possible by only one molecular target. For both *Leuconostoc* strains (1113/11 and 3696/08), the other set of primers for *tuf* gene amplification had to be used [60].

Strain 3696/08 was correctly identified as *L. citreum* by 16S rRNA, *tuf*, and *rpoB* gene sequencing, but amplification of the *sodA* gene was problematic. The primers d1 and d2 [24] used for the *sodA* gene in other *Streptococcus* strains did not result in PCR product amplification.

In our study, *S. pseudopneumoniae* was not identified by any of the four Sanger sequencing-based or phenotypic methods. Arbique et al. and Harf-Monteil et al. observed similarity between the isolates identified as *S. pseudopneumoniae* and *S. pneumoniae*, which demonstrated a high degree of homology and shared phenotypic characteristics [61,62]. However, in 2011, Zbinden et al. suggested that sequencing of the *recA* gene could differentiate between *S. pneumoniae* and *S. pseudopneumoniae* [31]. In our study, in addition to identification of the *Streptococcus mitis* group, we used Sanger sequencing of the *recA* gene, which successfully confirmed the identities of the *S. pseudopneumoniae*, *S. pneumoniae*, *S. mitis*, *S. oralis*, and *S. infantis* species. Moreover, it was the only method that correctly identified the p41 strain as *S. pseudopneumoniae*.

In *Streptococcus* species genetic diagnostics, other molecular target such as sequencing of the *ddl* or *gdh* genes could also be used [63,64]. However, these targets are not commonly used and are usually used for identification of specific species groups [65,66]. The advanced molecular diagnostics precision should definitely be strengthened with methods based on next-generation sequencing, but the costs and challenging data analysis are the pitfalls of these methods to be used in routine diagnostic laboratories [67].

To conclude, phenotypic methods such as the Vitek^®^ 2 system and MALDI-TOF MS constitute basic methods because the results are received after approximately 8 h and are characterized by lower costs than those of genetic methods. However, Sanger sequencing and PCR-based approaches proved to be excellent tools for identification at the species level for both *Streptococcus* and *Enterococcus* strains. We also proved that the use of only one method is often not enough for appropriate identification at the species level.

## 4. Materials and Methods

### 4.1. Ethical Approval

This article does not contain any studies with human participants or animals performed by any of the authors.

### 4.2. Bacterial Isolates

The collection of bacterial isolates included 30 isolates of 15 *Streptococcus* species, five *Enterococcus* species, two *Leuconostoc* species, and one isolate each from *Globicatella* and *Granulicatella* species recovered from clinical origin (Table 4). Most isolates were recovered from the National Medicines Institute in Warsaw (*n* = 13), with five isolates from the University Medical Center Groningen and 12 from Pescara Local Hospital. The isolates were cultured on blood agar medium with 5% sheep blood (bioMérieux, La Balme Les Grottes, France) and incubated at 37 °C in an atmosphere of 5% CO_2_ for 20 h.

### 4.3. Phenotypic Identification Tests

All isolates were identified using two phenotypic tests. The Vitek^®^ 2 system (bioMérieux, La Balme Les Grottes, France) was used to identify isolates at the genus and species levels. The suspension used in the Vitek^®^ 2 system was adjusted to a McFarland standard of 0.5 by using a densitometer and interpreted according to the manufacturer’s instructions. A score of ≥96% indicated excellent species identification; 91–95% indicated very good species identification. A score of 89–92% indicated good species identification. For streptococcal serotype identification, the Pastorex™ Strep Test Kit (Bio-Rad, Hercules, CA, USA) was used. The bacterial cells were suspended in 300 μL of enzymatic extract and incubated at 37 °C for 15 min. After incubation, the reagent with antibodies and bacterial suspension was applied to identification cards and mixed. The results were read after 30 s.

### 4.4. MALDI-TOF MS Identification

The MicroFlex MALDI-TOF mass spectrometer with MALDI Biotyper software 2.0 (Bruker Daltonics, Bremen, Germany) was used for isolate identification. Identification of isolates PL434, 1113/11, 3696/08, p41, and 1375/11 using MALDI-TOF MS was performed by The Microbiological Laboratory of the Jagiellonian Center of Innovation (Krakow, Poland). Sample extraction and strain identification were performed following the manufacturer’s instructions. A score of >2 indicated correct genus and probable species identification.

### 4.5. Genomic DNA Extraction

The Qiagen DNeasy Blood & Tissue Kit (Qiagen, Germantown, MD, USA) was used for genomic DNA extraction. Bacteria were homogenized with a TissueLyser II (Qiagen, Germantown, MD, USA) for five minutes at a frequency of 50 Hz. After homogenization, the tubes were centrifuged for 10 min at 13 200 rpm. The subsequent steps were performed according to the manufacturer’s instructions.

### 4.6. PCR Amplification of the 16S rRNA, sodA, rpoB tuf, and recA Genes

Both bacterial DNA and the negative control (nuclease-free H_2_O (EurX—Molecular Biology Products, Gdansk, Poland)) were amplified with primers for a given locus. As shown in Table 5 and Table 6, primers specific for the targeted locus were used as described previously [21,24,25,26,31,60,68]. Based on our previous studies, the PCR programs were modified slightly to obtain increased product quality [13].

All PCR products were resolved by electrophoresis in 1% agarose with 1× TAE and then purified using the DNA Clean & Concentrator™ Kit (Zymo Research, Irvine, CA, USA; A&A Biotechnology, Gdynia, Poland). Concentrations and purity were measured using a NanoDrop ND-1000. Sanger sequencing was performed at GATC Eurofins Genomics (Ebersberg, Germany) and Genomed S.A. (Warsaw, Poland) with the same primers as those used for PCR (Table 5 and Table 6).

### 4.7. Sanger Sequencing Analysis of the 16S rRNA, sodA, rpoB, and tuf Genes

The Sanger sequencing results were analyzed using Chromas software (version: 2.6.6). Nucleotide BLAST (Basic Local Alignment Search Tool http://www.ncbi.nlm.nih.gov/BLAST/) was used to analyze the obtained sequences and align them to the reference sequences deposited in the GenBank (https://www.ncbi.nlm.nih.gov/nucleotide/) and leBIBI^QBPP^ (leBIBI-Quick BioInformatic Phylogeny of Prokaryotes) (https://umr5558-bibiserv.univ-lyon1.fr/lebibi/lebibi.cgi) databases. The first and second best species alignments were analyzed. To identify the selected strain at the species level, the criterion of ≥99% first best match with the reference database and a difference of at least two nucleotides between the first and second best matches was used [13,69]. All sequences were aligned in ClustalW. The phylogenetic trees were constructed using the neighbor-joining method. The percentage of replicate trees in which the associated taxa clustered together in the bootstrap test (1000 replicates) and evolutionary distances were computed using the Jukes–Cantor method (MEGA, version 7.0.26, Pennsylvania State University, State College, PA, USA). Pairwise comparison of each pair of sequences was performed using CLC Genomics Workbench (version 8.1, Qiagen, USA).

### 4.8. Sanger Sequencing Analysis of the recA Gene

The obtained *recA* gene sequences (313 bp) for the S63, PL427, PL431, p63, and p41 strains were analyzed at six specific nucleotide positions (97, 160, 199, 247, 250, and 280). For precise differentiation of species within the mitis complex, the reference sequences of the *recA* genes from *S. pneumoniae* NCTC 7465, *Streptococcus mitis* NCTC 12261, *Streptococcus oralis* NCTC 11427, *Streptococcus pseudopneumoniae* ATCC BAA-960, and *Streptococcus infantis* ATCC 700779 (reference numbers in GenBank: HM572273, HM572275, HM572276, HM572274, HM572277, respectively) were used [25].

### 4.9. Nucleotide Sequence Accession Numbers

The 124 sequences for 21 *Streptococcus*, five *Enterococcus*, and four other species were annotated using the NCBI BankIt tool and deposited in the GenBank database (https://www.ncbi.nlm.nih.gov/genbank/) under the following accession numbers: for the 16S rRNA gene, MT535599-MT535603, MT535764 and MT535859-MT535882; for the *sodA* gene, MT560910-MT560938; for the *tuf* gene, MT560846-MT560874 and MT707819; for the *rpoB* gene, MT560875-MT560904; and for the *recA* gene, MT560905-MT560909.

## Figures and Tables

**Table 1:** Performance of the serotyping of the Vitek^®^ 2 system and MALDI-TOF MS identification of *Streptococcus* spp. and *Enterococcus* spp.

Isolate No.	Serotype	Species	Vitek^®^ 2 System Results		MALDI-TOF MS
			Probability of Identification	Discrimination Level	Species (Score Values)
E15	G	*Enterococcus avium*	95%	Very good	not performed
E2	D	*Enterococcus casseliflavus*	98%	Excellent	not performed
E5	AGL	*Enterococcus durans*	94%	Very good	not performed
E28	AGL	*Enterococcus faecalis*	99%	Excellent	not performed
E10	D	*Enterococcus raffinosus*	94%	Very good	not performed
1375/11	-	*Globicatella sanguinis*	96%	Excellent	*Globicatella sanguinis* (2.39)
PL434	D	*Kocuria rosea*	90%	Good	*Granulicatella adiacens* (2.22)
3696/08	D	*Leuconostoc citreum*	97%	Excellent	*Leuconostoc citreum* (2.10)
1113/11	AGL	*Leuconostoc lactis*	97%	Excellent	*Leuconostoc lactis* (2.20)
S19	B	*Streptococcus agalactiae*	97%, 99%	Excellent	not performed
4734/08	C	*Streptococcus anginosus*	97%	Excellent	not performed
5898/10	AGL	*Streptococcus anginosus*	95%	Very good	not performed
1816/15	-	*Streptococcus mitis/oralis*	99%	Excellent	not performed
1226/14	-	*Streptococcus mitis/oralis*	95%	Very good	not performed
1107/09	C	*Streptococcus gordonii*	97%	Excellent	not performed
6922/09	C	*Streptococcus anginosus/gordonii*	96%	Low	not performed
1860/08	G	*Streptococcus* *agalactiae/* *dysgalactiae*	98%	Low	not performed
8190/10	G	*Streptococcus dysgalactiae*	96%	Excellent	not performed
5010/12	AGL	*Streptococcus gallolyticus*	99%	Excellent	not performed
S18	AGL	*Streptococcus gallolyticus*	97%	Excellent	not performed
PL427	-	*Streptococcus mitis/oralis*	99%	Excellent	not performed
PL428	-	*Streptococcus lutetiensis*	97%	Excellent	not performed
PL431	D	*Streptococcus mitis/oralis*	90%	Good	not performed
S16	-	*Streptococcus parasanguinis*	99%	Excellent	not performed
S63	C	*Streptococcus mitis/oralis*	98%	Excellent	not performed
1374/11	-	*Streptococcus mitis/oralis*	99%	Excellent	not performed
p63	C	*Streptococcus pneumoniae*	99%	Excellent	not performed
p41	D	×	×	×	*Streptococcus pneumoniae* (2.08)
S47	A	*Streptococcus pyogenes*	95%	Very good	not performed
PL433	G	*Streptococcus salivarius*	96%	Excellent	not performed

AGL—agglutination; All ambiguous *Streptococcus*, *Enterococcus* and nonstreptococcus species are indicated in a dark red color. ×—lack of identification.

**Table 2:** Summary of the species identification based on 16S rRNA, *sodA*, *tuf*, and *rpoB* genes.

Identified Species	Isolate No.	16S rRNA Gene	*sodA* Gene	*tuf* Gene	*rpoB* Gene
***Enterococcus avium***	E15	*Enterococcus avium*	*Enterococcus avium*	*Enterococcus avium*	*Enterococcus avium*
***Enterococcus casseliflavus***	E2	*Enterococcus casseliflavus*	*Enterococcus casseliflavus*	*Enterococcus casseliflavus*	*Enterococcus casseliflavus*
***Enterococcus durans***	E5	*Enterococcus durans*	*Enterococcus durans*	*Enterococcus durans*	*Enterococcus durans*
***Enterococcus faecalis***	E28	*Enterococcus faecalis*	*Enterococcus faecium*	*Enterococcus faecalis*	*Enterococcus faecalis*
***Enterococcus raffinosus***	E10	*Enterococcus raffinosus/gilvus*	*Enterococcus raffinosus*	*Enterococcus raffinosus*	*Enterococcus raffinosus*
***Globicatella sanguinis***	1375/11	*Globicatella sanguinis*	*Globicatella sanguinis*	*Globicatella sanguinis*	*Globicatella sanguinis*
***Granulicatella adiacens***	PL434	*Granulicatella adiacens*	*Granulicatella adiacens*	*Granulicatella adiacens*	*Granulicatella adiacens*
***Leuconostoc citreum***	3696/08	*Leuconostoc citreum*	no amplification product	*Leuconostoc citreum*	*Leuconostoc citreum*
***Leuconostoc*** ***lactis***	1113/11	*Leuconostoc lactis /garlicum*	*×*	*Leuconostoc lactis /garlicum*	*Leuconostoc lactis*
***Streptococcus*** ***agalactiae***	S19	*Streptococcus agalactiae*	*Streptococcus agalactiae*	*Streptococcus agalactiae*	*Streptococcus agalactiae*
***Streptococcus anginosus***	4734/08	*Streptococcus anginosus*	*Streptococcus anginosus*	*Streptococcus anginosus*	*Streptococcus anginosus*
***Streptococcus anginosus***	5898/10	*Streptococcus anginosus*	*Streptococcusanginosus/milleri*	*Streptococcus anginosus/milleri*	*Streptococcus anginosus/ intermedius*
***Streptococcus australis***	1816/15	*Streptococcus australis*	*Streptococcus australis*	*Streptococcus australis*	*Streptococcus australis*
***Streptococcus australis***	1226/14	*Streptococcus australis/sanguinis*	*Streptococcus australis*	*Streptococcus australis*	*Streptococcus australis*
***Streptococcus constellatus***	1107/09	*Streptococcus constellatus*	*Streptococcus anginosus/milleri*	*Streptococcus anginosus/milleri*	*Streptococcus constellatus*
***Streptococcus constellatus***	6922/09	*Streptococcus constellatus*	*Streptococcus anginosus/milleri*	*Streptococcus anginosus/milleri*	*Streptococcus constellatus*
***Streptococcus dysgalactiae***	1860/08	*Streptococcus dysgalactiae*	*Streptococcus dysgalactiae*	*Streptococcus dysgalactiae*	*Streptococcus dysgalactiae*
***Streptococcus dysgalactiae***	8190/10	*Streptococcus dysgalactiae*	*Streptococcus dysgalactiae*	*Streptococcus dysgalactiae*	*Streptococcus dysgalactiae*
***Streptococcus gallolyticus***	5010/12	*Streptococcus gallolyticus*	*Streptococcus gallolyticus*	*Streptococcus gallolyticus*	*Streptococcus gallolyticus*
***Streptococcus gallolyticus***	S18	*Streptococcus gallolyticus*	*Streptococcus gallolyticus*	*Streptococcus gallolyticus*	*Streptococcus gallolyticus*
***Streptococcus infantis***	PL427	*Streptococcus infantis*	*Streptococcus infantis*	*Streptococcus oralis*	*Streptococcusaustralis/infantis*
***Streptococcus*** ***lutetiensis***	PL428	*Streptococcus lutetiensis*	*Streptococcus lutetiensis/* *infantarius*	*Streptococcus lutetiensis/ infantarius*	*Streptococcus lutetiensis*
***Streptococcus mitis***	PL431	*Streptococcus mitis*	*Streptococcus mitis*	*Streptococcus mitis*	*Streptococcus mitis*
***Streptococcus oralis***	S16	*Streptococcus oralis*	*Streptococcus oralis*	*Streptococcus oralis/infantis*	*Streptococcus oralis*
***Streptococcus oralis***	S63	*Streptococcus oralis*	*Streptococcus oralis*	*Streptococcus oralis/infantis*	*Streptococcus oralis*
***Streptococcus parasanguinis***	1374/11	*Streptococcus parasanguinis*	*Streptococcus parasanguinis/oralis*	*Streptococcus parasanguinis*	*Streptococcus parasanguinis*
***Streptococcus pneumoniae***	p63	*Streptococcus pneumoniae*	*Streptococcus pneumoniae*	*Streptococcus pneumoniae*	*Streptococcus pneumoniae*
***Streptococcus pseudopneumoniae***	p41	*Streptococcus pneumoniae/mitis*	*Streptococcus mitis/cristatus*	*Streptococcus pneumoniae/mitis*	*Streptococcus pseudopneumoniae/mitis*
***Streptococcus pyogenes***	S47	*Streptococcus pyogenes*	*Streptococcus pyogenes/dysgalactiae*	*Streptococcus pyogenes*	*Streptococcus pyogenes*
***Streptococcus salivarius***	PL433	*Streptococcus salivarius*	*Streptococcus salivarius*	*Streptococcus salivarius*	*Streptococcus salivarius*

All ambiguous *Streptococcus*, *Enterococcus* and nonstreptococcal species are indicated in dark red color. ×—lack of reference sequences.

**Table 3:** Summary of the performance of 16S rRNA, *sodA*, *tuf*, and *rpoB* genes sequencing used for differentiation of *Streptococcus* and *Enterococcus* genera.

*Streptococcus* spp. (*n* = 21 Strains) *Enterococcus* spp. (*n* = 5 Strains)			Unambiguous Species Identification	No Identification at the Species Level
**Sanger Sequencing**	16S rRNA gene	*Enterococcus*	4 (80%)	1 (20%)
		*Streptococcus*	19 (90%)	2(10%)
	*sodA* gene	*Enterococcus*	4 (80%)	1 (20%)
		*Streptococcus*	12 (57%)	9 (43%)
	*tuf* gene	*Enterococcus*	5 (100%)	0 (0%)
		*Streptococcus*	13 (62%)	8 (38%)
	*rpoB* gene	*Enterococcus*	5 (100%)	0 (0%)
		*Streptococcus*	18 (86%)	3 (14%)

**Table 4:** *Streptococcus* and *Enterococcus* species used for analyses.

	Species	Isolate Number
*Enterococcus* *n* = 5	*Enterococcus avium*	E15 ^c^
	*Enterococcus casseliflavus*	E2 ^c^
	*Enterococcus durans*	E5 ^c^
	*Enterococcus faecalis*	E28 ^c^
	*Enterococcus raffinosus*	E10 ^c^
Other species *n* = 4	*Globicatella sanguinis*	1375/11 ^a^
	*Granulicatella adiacens*	PL434 ^b^
	*Leuconostoc citreum*	3696/08 ^a^
	*Leuconostoc lactis*	1113/11 ^a^
*Streptococcus* *n* = 21	*Streptococcus agalactiae*	S19 ^c^
	*Streptococcus anginosus*	4734/08 ^a^
		5898/10 ^a^
	*Streptococcus australis*	1816/15 ^a^
		1226/14^a^
	*Streptococcus constellatus*	1107/09 ^a^
		6922/09 ^a^
	*Streptococcus dysgalactiae*	1860/08 ^a^
		8190/10 ^a^
	*Streptococcus gallolyticus*	5010/12 ^a^
		S18 ^c^
	*Streptococcus infantis*	PL427 ^b^
	*Streptococcus lutetiensis*	PL428 ^b^
	*Streptococcus mitis*	PL431 ^b^
	*Streptococcus oralis*	S16 ^c^
		S63 ^c^
	*Streptococcus parasanguinis*	1374/11 ^a^
	*Streptococcus pneumoniae*	p63 ^c^
	*Streptococcus pseudopneumoniae*	p41 ^c^
	*Streptococcus pyogenes*	S47 ^c^
	*Streptococcus salivarius*	PL433 ^b^

**^a^** National Medicines Institute in Warsaw; **^b^** University Medical Center Groningen; **^c^** Pescara Local Hospital–Italy.

**Table 5:** Oligonucleotide sequencing primers and PCR program used for amplification of 16S rRNA, *sodA*, *tuf*, *rpoB* and *recA* genes.

Target Gene	PCR Program	Cycles (Steps 2–4)	Amplification Primers (5′→3′)	Amount of Sequenced PCR Product	Reference
16S rRNA (1284-bp)	94 °C for 2 min 94 °C for 30 s 58 °C for 30 s 72 °C for 1 min 72 °C for 5 min	25	LPW57 AGTTTGATCCTGGCTCAG	250 ng	[68]
			LPW58 AGGCCCGGGAACGTATTCAC		
*sodA* (430-bp)	95 °C for 3 min 95 °C for 30 s 43 °C for 1 min **^A^** 72 °C for 1 min 30 s 72 °C for 10 min	35	d1 CCITAYICITAYGAYGCIYTIGARCC	100 ng	[24]
			d2 ARRTARTAIGCRTGYTCCCAIACRTC		
*tuf* (830-bp)	95 °C for 2 min 94 °C for 30 s 50 °C for 30 s **^B^** 72 °C for 1 min 30 s 72 °C for 10 min	30	tuf-F CCAATGCCACAAACTCGT	200 ng	[25]
			tuf-R CCTGAACCAACAGTACGT		
*rpoB* (860-bp)	95 °C for 2 min 94 °C for 30 s 52 °C for 30 s 72 °C for 1 min 72 °C for 5 min	35	Strepto-F AARYTIGGMCCTGAAGAAAT	200 ng	[26]
			Strepto-R TGIARTTTRTCATCAACCATGTG		
*recA* (850-bp)	95 °C for 2 min 94 °C for 30 s 54 °C for 30 s 72 °C for 1 min 30 s 72 °C for 10 min	30	RStrGseq81 GAAAWWIATYGARAAAGAITTTGGTAA	150 ng	[31]
			RStrGseq937 TTYTCAGAWCCTTGICCAATYTTYTC		

**^A^ 40 °C** (strains: S18, S19, 6922/08, 1226/14, 5898/10, PL434, PL434, E10) **45 °C** (strain: 1860/08) **50 °C** (strains: S16, 1816/15, 8190/10) **52 °C** (strain: p41) **^B^** **53 °C** (strains: 1226/14, PL427) **59 °C** (strains: E10, E15).

**Table 6:** Alternative oligonucleotide sequencing primers and PCR program used for amplification of *sodA* and *tuf* genes.

Target Gene	PCR Program	Cycles (Steps 2–4)	Amplification Primers (5′→3′)	Amount of Sequenced PCR Product	Reference
*sodA* (430-bp)	94 °C for 5 min 94 °C for 30 s 50 °C for 1 min **^C^** 72 °C for 30 s 72 °C for 5 min	30	sodA-F TRCAYCATGAYAARCACCAT	100 ng	[21]
			sodA-R ARRTARTAMGCRTGYTCCCARACRTC		
*tuf* (830-bp)	95 °C for 3 min 95 °C for 30 s 55 °C for 30 s **^D^** 72 °C for 1 min 72 °C for 7 min	35	U1 AAYATGATIACIGGIGCIGCICARATGGA	200 ng	[60]
			U2 AYRTTITCICCIGGCATIACCAT		

**^C^50 °C** (strain: 3696/08) **^D^ 55 °C** (strains: 1113/11, 3696/08).

## References

[1] Lal D., Verma M., Lal R. (2011). Exploring internal features of 16S rRNA gene for identification of clinically relevant species of the genus *Streptococcus*. Ann. Clin. Microbiol. Antimicrob..

[2] Kohler W. (2007). The present state of species within the genera *Streptococcus* and *Enterococcus*. Int. J. Med. Microbiol..

[3] Gao X.Y., Zhi X.Y., Li H.W., Klenk H.P., Li W.J. (2014). Comparative genomics of the bacterial genus *Streptococcus* illuminates evolutionary implications of species groups. PLoS ONE.

[4] Farley M.M., Strasbaugh L.J. (2001). Group B streptococcal disease in nonpregnant adults. Clin. Infect. Dis..

[5] Henriques-Normark B., Tuomanen E.I. (2013). The pneumococcus: Epidemiology, microbiology, and pathogenesis. Cold Spring Harb. Perspect. Med..

[6] Pekuz S., Soysal A., Akkoc G., Atıcı S., Yakut N., Gelmez G.A., Kadayifci E.K., Güneser D., Demir S.O., Söyletir G. (2019). Prevalence of nasopharyngeal carriage, serotype distribution, and antimicrobial resistance of *Streptococcus pneumoniae* among children with chronic diseases. Jpn. J. Infect. Dis..

[7] Gerhardt P., Murray R.G.E., Willis A., Krieg N.R. (1994). Methods for General and Molecular Bacteriology.

[8] Kawamura Y., Hou X.G., Sultana F., Miura H., Ezaki T. (1995). Determination of 16S rRNA sequences of *Streptococcus mitis* and *Streptococcus gordonii* and phylogenetic relationships among members of the genus *Streptococcus*. Int. J. Syst. Bacteriol..

[9] Facklam R., Elliott J.A. (1995). Identification, classification, and clinical relevance of catalase-negative, gram-positive cocci, excluding the streptococci and enterococci. Clin. Microbiol. Rev..

[10] Parks T., Barrett L., Jones N. (2015). Invasive streptococcal disease: A review for clinicians. Br. Med. Bull..

[11] Schleifer K.H., Kilpper-Balz R. (1984). Transfer of *Streptococcus faecalis* and *Streptococcus faecium* to the genus *Enterococcus* nom. rev. as *Enterococcus faecalis* comb. nov. and *Enterococcus faecium* comb. nov. Int. J. Syst. Evol..

[12] Devriese L., Baele M., Butaye P. (2006). The Genus Enterococcus. The Prokaryotes.

[13] Kosecka-Strojek M., Sabat A.J., Akkerboom V., Kooistra-Smid A.M.D.M., Miedzobrodzki J., Friedrich A.W. (2019). Development of a reference data set for assigning *Streptococcus* and *Enterococcus* species based on next generation sequencing of the 16S-23S rRNA region. Antimicrob Resist. Infect. Control.

[14] Savini V., Franco A., Gherardi G., Marrollo R., Argentieri A.V., De Araujo F.P., Amoruso R., Battisti A., Fazii P., Carretto E. (2014). Beta-hemolytic, multi-lancefield antigen-agglutinating *Enterococcus durans* from a pregnant woman, mimicking *Streptococcus agalactiae*. J. Clin. Microbiol..

[15] Ferede Z.T., Tullu K.D., Derese S.G., Yeshanew A.G. (2018). Prevalence and antimicrobial susceptibility pattern of *Enterococcus* species isolated from different clinical samples at Black Lion Specialized Teaching Hospital, Addis Ababa, Ethiopia. BMC Res. Notes.

[16] Top J., Willems R., Bonten M. (2008). Emergence of CC17 *Enterococcus faecium*: From commensal to hospital-adapted pathogen. FEMS Immunol. Med. Microbiol..

[17] Fernández-Hidalgo N., Escolà-Vergé L. (2019). *Enterococcus faecalis* Bacteremia: Consider an echocardiography, but consult an infectious diseases specialist. J. Am. Coll. Cardiol..

[18] Ligozzi M., Bernini C., Bonora M.G., De Fatima M., Zuliani J., Fontana R. (2002). Evaluation of the VITEK 2 system for identification and antimicrobial susceptibility testing of medically relevant gram-positive cocci. J. Clin. Microbiol..

[19] Chen J.H., She K.K., Wong O.Y., Teng J.L., Yam W.C., Lau S.K., Woo P.C., Cheng V.C., Yuen K.Y. (2015). Use of MALDI Biotyper plus ClinProTools mass spectra analysis for correct identification of *Streptococcus pneumoniae* and *Streptococcus mitis*/*oralis*. J. Clin. Pathol..

[20] Singhal N., Kumar M., Kanaujia P.K., Virdi J.S. (2015). MALDI-TOF mass spectrometry: An emerging technology for microbial identification and diagnosis. Front. Microbiol..

[21] Hoshino T., Fujiwara T., Kilian M. (2005). Use of phylogenetic and phenotypic analyses to identify nonhemolytic streptococci isolated from bacteremic patients. J. Clin. Microbiol..

[22] Isaksson J., Rasmussen M., Nilson B., Stadler L.S., Kurland S., Olaison L., Ek E., Herrmann B. (2015). Comparison of species identification of endocarditis associated viridans streptococci using *rnpB* genotyping and 2 MALDI-TOF systems. Diagn. Microbiol. Infect. Dis..

[23] Clarridge J.E. (2004). Impact of 16S rRNA gene sequence analysis for identification of bacteria on clinical microbiology and infectious diseases. Clin. Microbiol. Rev..

[24] Poyart C., Quesne G., Coulon S., Berche P., Trieu-Cuot P. (1998). Identification of streptococci to species level by sequencing the gene encoding the manganese-dependent superoxide dismutase. J. Clin. Microbiol..

[25] Li X., Xing J., Li B., Wang P., Liu J. (2012). Use of *tuf* as a target for sequence-based identification of Gram-positive cocci of the genus *Enterococcus*, *Streptococcus*, coagulase-negative *Staphylococcus*, and *Lactococcus*. Ann. Clin. Microbiol. Antimicrob..

[26] Drancourt M., Roux V., Fournier P.E., Raoult D. (2004). *rpoB* gene sequence-based identification of aerobic Gram-positive cocci of the genera *Streptococcus*, *Enterococcus*, *Gemella*, *Abiotrophia*, and *Granulicatella*. J. Clin. Microbiol..

[27] Jensen A., Scholz C.F.P., Kilian M. (2016). Re-evaluation of the taxonomy of the Mitis group of the genus *Streptococcus* based on whole genome phylogenetic analyses, and proposed reclassification of *Streptococcus dentisani* as *Streptococcus oralis* subsp. *dentisani* comb. nov., *Streptococcus tigurinus* as Streptococcus *oralis* subsp. *tigurinus* comb. nov., and *Streptococcus oligofermentans* as a later synonym of *Streptococcus cristatus*. Int. J. Syst. Evol. Microbiol..

[28] Sadowy E., Hryniewicz W. (2020). Identification of *Streptococcus pneumoniae* and other Mitis streptococci: Importance of molecular methods. Eur. J. Clin. Microbiol. Infect. Dis..

[29] Dekker J.P., Lau A.F. (2016). An Update on the *Streptococcus bovis* Group: Classification, Identification, and Disease Associations. J. Clin. Microbiol..

[30] Werno A.M., Christner M., Anderson T.P., Murdoch D.R. (2012). Differentiation of *Streptococcus pneumoniae* from nonpneumococcal streptococci of the *Streptococcus mitis* group by Matrix-Assisted Laser Desorption Ionization–Time of Flight Mass Spectrometry. J. Clin. Microbiol..

[31] Zbinden A., Köhler N., Bloemberg G.V. (2011). *recA*-based PCR assay for accurate differentiation of *Streptococcus pneumoniae* from other viridans streptococci. J. Clin. Microbiol..

[32] (2017). BIO-RAD. 2017. Manufacturer’s Instructions: Agglutination Test for the Grouping Streptococci Belonging to Groups A, B, C, D, F, G. PASTOREX™ STREP. https://www.biorad.com/webroot/web/pdf/inserts/CDG/en/61721_880999_EN.pdf.

[33] Gherardi G., Angeletti S., Panitti M., Pompilio A., Di Bonaventura G., Crea F., Avola A., Fico L., Palazzo C., Sapia G.F. (2012). Comparative evaluation of the Vitek-2 Compact and Phoenix systems for rapid identification and antibiotic susceptibility testing directly from blood cultures of Gram-negative and Gram-positive isolates. Diagn. Microbiol. Infect. Dis..

[34] de Cueto M., Ceballos E., Martinez-Martinez L., Perea E.J., Pascual A. (2004). Use of positive blood cultures for direct identification and susceptibility testing with the Vitek 2 system. J. Clin. Microbiol..

[35] Chen J., Lee S., Yang B., Lu J. (2008). Rapid identification and susceptibility testing using the VITEK 2 system using culture fluids from positive BacT/ALERT blood cultures. J. Microbiol. Immunol. Infect..

[36] Romero-Gómez M.P., Gómez-Gil R., Paño-Pardo J.R., Mingorance J. (2012). Identification and susceptibility testing of microorganism by direct inoculation from positive blood culture bottles by combining MALDI-TOF and Vitek-2 Compact is rapid and effective. J. Infect..

[37] Dubois D., Segonds C., Prere M.F., Marty N., Oswald E. (2013). Identification of clinical *Streptococcus pneumoniae* isolates among other alpha and nonhemolytic streptococci by use of the Vitek MS matrix-assisted laser desorption ionization-time of flight mass spectrometry system. J. Clin. Microbiol..

[38] Monteiro J., Inoue F.M., Lobo A.P., Sugawara E.K., Boaretti F.M., Tufik S. (2015). Fast and reliable bacterial identification direct from positive blood culture using a new TFA sample preparation protocol and the Vitek^®^ MS system. J. Microbiol. Methods.

[39] Febbraro F., Rodio D.M., Puggioni G., Antonelli G., Pietropaolo V., Trancassini M. (2016). MALDI-TOF MS Versus VITEK^®^2: Comparison of systems for the identification of microorganisms responsible for bacteremia. Curr. Microbiol..

[40] Srinivasan R., Karaoz U., Volegova M., MacKichan J., Kato-Maeda M., Miller S., Nadarajan R., Brodie E.L., Lynch S.V. (2015). Use of 16S rRNA Gene for identification of a broad range of clinically relevant bacterial pathogens. PLoS ONE.

[41] Janda M.J., Sharon L.A. (2007). 16S rRNA gene sequencing for bacterial identification in the diagnostic laboratory: Pluses, Perils, and Pitfalls. J. Clin. Microbiol..

[42] Summanen P.H., Rowlinson M., Wooton J., Finegold S.M. (2009). Evaluation of genotypic and phenotypic methods for differentiation of the members of the Anginosus group streptococci. Eur. J. Clin. Microbiol. Infect. Dis..

[43] Stackebrandt E., Frederiksen W., Garrity G.M., Grimont P., Kämpfer P.A.D., Maiden M.C.J., Nesme X., Rosselló-Mora R., Swings J., Trüper H.G. (2002). Report of the ad hoc committee for the re-evaluation of the species definition in bacteriology. Int. J. Syst. Evol. Microbiol..

[44] Lerat E., Daubin V., Ochman H., Moran N.A. (2005). Evolutionary origins of genomic repertoires in bacteria. PLoS Biol..

[45] Leray M., Knowlton N., Ho S.L., Nguyen B.N., Machida R.J. (2019). GenBank is a reliable resource for 21st century biodiversity research. Proc. Natl. Acad. Sci. USA.

[46] Zbinden A., Mueller N.J., Tarr P.E., Spröer C., Keller P.M., Bloemberg G.V. (2012). *Streptococcus tigurinus* sp. nov., isolated from blood of patients with endocarditis, meningitis and spondylodiscitis. Int. J. Syst. Evol. Microbiol..

[47] Poyart C., Quesne G., Trieu-Cuot P. (2002). Taxonomic dissection of the *Streptococcus bovis* group by analysis of manganese-dependent superoxide dismutase gene (*sodA*) sequences: Reclassification of *‘Streptococcus infantarius* subsp. *coli’* as *Streptococcus lutetiensis* sp. nov. and of *Streptococcus bovis* biotype 11.2 as *Streptococcus pasteurianus* sp. nov. Int. J. Syst. Evol. Microbiol..

[48] Jackson C.R., Fedorka-Cray P.J., Barrett J.B. (2004). Use of a genus- and species-specific multiplex PCR for identification of enterococci. J. Clin. Microbiol..

[49] Castillo-Rojas G., Mazari-Hiríart M., de León S.P., Amieva-Fernández R.I., Agis-Juárez R.A., Huebner J., López-Vidal Y. (2013). Comparison of *Enterococcus faecium* and *Enterococcus faecalis* strains isolated from water and clinical samples: Antimicrobial susceptibility and genetic relationships. PLoS ONE.

[50] Clarridge J.E., Osting C., Jalali M., Osborne J., Waddington M. (1999). Genotypic and phenotypic characterization of “*Streptococcus milleri*” group isolates from a Veterans Administration hospital population. J. Clin. Microbiol..

[51] Arinto-Garcia R., Pinho M.D., Carriço J.A., Melo-Cristino J., Ramirez M. (2015). Comparing Matrix-Assisted Laser Desorption Ionization-Time of Flight Mass Spectrometry and phenotypic and molecular methods for identification of species within the *Streptococcus anginosus* Group. J. Clin. Microbiol..

[52] Limia A., Alarcón T., Jiménez M.L., López-Brea M. (2000). Comparison of three methods for identification of *Streptococcus milleri* group isolates to species level. Eur. J. Clin. Microbiol. Infect. Dis..

[53] Collins M.D., Aguirre M., Facklam R.R., Shallcross J., Williams A.M. (1992). *Globicatella sanguis* gen.nov., sp.nov., a new gram-positive catalase-negative bacterium from human sources. J. Appl. Bacteriol..

[54] Shewmaker P.L., Steigerwalt A.G., Shealey L., Weyant R., Facklam R. (2001). DNA relatedness, phenotypic characteristics, and antimicrobial susceptibilities of *Globicatella sanguinis* strains. J. Clin. Microbiol..

[55] Miller A.O., Buckwalter S.P., Henry M.W., Wu F., Maloney K.F., Abraham B.K., Hartman B.J., Brause B.D., Whittier S., Walsh T.J. (2017). *Globicatella sanguinis* Osteomyelitis and Bacteremia: Review of an emerging human pathogen with an expanding spectrum of disease. Open Forum Infect. Dis..

[56] Kawamura Y., Hou X.G., Sultana F., Liu S., Yamamoto H., Ezaki T. (1995). Transfer of *Streptococcus adjacens* and *Streptococcus defectivus* to *Abiotrophi*a gen. nov. as *Abiotrophia adiacens* comb. nov. and *Abiotrophia defectiva* comb. nov., respectively. Int. J. Syst. Bacteriol..

[57] Collins M.D., Lawson P.A. (2000). The genus *Abiotrophia* (Kawamura et al.) is not monophyletic: Proposal of *Granulicatella* gen. nov., *Granulicatella adiacens* comb. nov., *Granulicatella elegans* comb. nov. and *Granulicatella balaenopterae* comb. nov. Int. J. Syst. Evol. Microbiol..

[58] Patel T., Molloy A., Smith R., Balakrishnan I. (2012). Successful treatment of *Leuconostoc* bacteremia in a neutropenic patient with tigecycline. Infect. Dis. Rep..

[59] Albanese A., Spanu T., Sali M., Novegno F., D’Inzeo T., Santangelo R. (2006). Molecular identification of *Leuconostoc mesenteroides* as a cause of brain abscess in an immunocompromised patient. J. Clin. Microbiol..

[60] Ke D., Picard F.J., Martineau F., Ménard C., Roy P.H., Ouellette M., Bergeron M.G. (1999). Development of a PCR assay for rapid detection of enterococci. J. Clin. Microbiol..

[61] Arbique J.C., Poyart C., Trieu-Cuot G., Quesne M., Carvalho G.S., Steigerwalt A.G., Morey R.E., Jackson D., Davidson R.J., Facklam R.R. (2004). Accuracy of phenotypic and genotypic testing for identification of *Streptococcus pneumoniae* and description of *Streptococcus pseudopneumoniae* sp. nov. J. Clin. Microbiol..

[62] Harf-Monteil C., Granello C., Le Brun C., Monteil H., Riegel P. (2006). Incidence and pathogenic effect of *Streptococcus pseudopneumoniae*. J. Clin. Microbiol..

[63] Nielsen X.C., Justesen U.S., Dargis R., Kemp M., Christensen J.J. (2009). Identification of clinically relevant nonhemolytic Streptococci on the basis of sequence analysis of 16S-23S intergenic spacer region and partial *gdh* gene. J. Clin. Microbiol..

[64] Garnier F., Gerbaud G., Courvalin P., Galimand M. (1997). Identification of clinically relevant viridans group streptococci to the species level by PCR. J. Clin. Microbiol..

[65] Harju I., Lange C., Kostrzewa M., Maier T., Rantakokko-Jalava K., Haanperä M. (2017). Improved differentiation of *Streptococcus pneumoniae* and other *S. mitis* group streptococci by MALDI Biotyper using an improved MALDI Biotyper database content and a novel result interpretation algorithm. J. Clin. Microbiol..

[66] Scott J.E., Li K., Filkins L.M., Zhu B., Kuchma S.L., Schwartzman J.D., O’Toole G.A. (2019). *Pseudomonas aeruginosa* can inhibit growth of streptococcal species via siderophore production. J. Bacteriol..

[67] Besser J., Carleton H.A., Gerner-Smidt P., Lindsey R.L., Trees E. (2018). Next-generation sequencing technologies and their application to the study and control of bacterial infections. Clin. Microbiol. Infect..

[68] Woo P.C., Leung A.S., Leung K.W., Yuen K.Y. (2001). Identification of slide coagulase positive, tube coagulase negative *Staphylococcus aureus* by 16S ribosomal RNA gene sequencing. Mol. Pathol..

[69] Sabat A.J., Van Zanten E., Akkerboom V., Wisselink G., van Slochteren K., de Boer R.F., Hendrix R., Friedrich A.W., Rossen J.W.A., Kooistra-Smid A.M.D. (2017). Targeted next-generation sequencing of the 16S-23S rRNA region for culture-independent bacterial identification-increased discrimination of closely related species. Sci. Rep..

